# Transmission Electron Microscopy as a Tool for the Characterization of Soft Materials: Application and Interpretation

**DOI:** 10.1002/advs.201600476

**Published:** 2017-01-31

**Authors:** Linda E. Franken, Egbert J. Boekema, Marc C. A. Stuart

**Affiliations:** ^1^ Electron Microscopy GroupGroningen Biomolecular Sciences and Biotechnology InstituteUniversity of GroningenNijenborgh 79747AGGroningenThe Netherlands; ^2^ Stratingh Institute for ChemistryUniversity of GroningenNijenborgh 79747AGGroningenThe Netherlands

**Keywords:** self‐assembly, vesicles, artefacts, sample preparation, cryo‐TEM

## Abstract

Transmission electron microscopy (TEM) provides direct structural information on nano‐structured materials and is popular as a characterization tool in soft matter and supramolecular chemistry. However, technical aspects of sample preparation are overlooked and erroneous image interpretations are regularly encountered in the literature. There are three most commonly used TEM methods as we derived from literature: drying, staining and cryo‐TEM, which are explained here with respect to their application, limitations and interpretation. Since soft matter chemistry relies on a lot of indirect evidence, the role of TEM for the correct evaluation of the nature of an assembly is very large. Mistakes in application and interpretation can therefore have enormous impact on the quality of present and future studies. We provide helpful background information of these three techniques, the information that can and cannot be derived from them and provide assistance in selecting the right technique for soft matter imaging. This essay warns against the use of drying and explains why. In general cryo‐TEM is by far the best suited method and many mistakes and over‐interpretations can be avoided by the use of this technique.

## 1 Introduction

For their urge to build more complex systems, supramolecular chemistry,^1^ dynamic combinatorial chemistry^2^ and systems chemistry^3^ rely on high‐quality TEM data.^4^, ^5^ The chemistry has evolved from single molecules towards molecular systems in which functionality and responsiveness are integrated in nano‐structured materials,^6^ for which TEM is an essential and powerful tool. It allows imaging of a large range of objects, from biological systems, e.g. cells^7^ or proteins,^8^, ^9^ to materials,^10^ aggregating of surfactants^11^, ^12^ and other self‐organising molecules into structures such as gels^13^ and vesicles.^14^ It is widely applied in both biology and organic and polymer chemistry. The increased demand for TEM data combined with easier access is leading to a growing number of inexperienced users who, lacking sufficient knowledge on possibilities and limitations of the technique, are contributing an increased number of scientific papers with application and interpretation errors.

From literature, the most frequently used TEM sample preparation techniques for soft matter were identified: drying, staining and cryo‐TEM. Surprisingly, in many studies samples are dried. Drying is a risky technique, well known to possibly alter the structure of soft nano‐objects and to give rise to aggregation of dissolved materials.^15^, ^16^ Although negative staining and cryo‐TEM are more suitable, many mistakes are made, some of which are called upon, but many go unnoticed. The interpretation of TEM data is often erroneous and in service of the hypothesis. This becomes apparent when, upon drying, similar structures appear sometimes black (high‐density material) and sometimes white (low‐density material) even within one figure. In this essay, each of the three approaches is demonstrated with doxorubicin‐loaded stealth liposomes^17^, ^18^ and amphiphilic nanotubes^4^, ^19^ and reviewed with respect to their possibilities and limitations for soft materials.

Many reviews have been published on artefacts in TEM, such as drying patterns,^20^, ^21^ incorrect focussing^22^, ^23^ and ice contamination.^16^, ^24^, ^25^ Good literature exists also about the inappropriate use of ‘quick‐freeze deep‐edge’ on colloidal suspensions^26^ and the over‐interpretation of stained images.^20^ However, mistakes are still made today as it remains unillustrated how important and relevant these papers are with respect to current literature. Especially in the field of soft matter chemistry, where TEM is usually but one of many measurements, papers that provide this type of TEM background information are overlooked. Also, the chemical orientation of the literature and the relatively small role that TEM takes in a typical supramolecular chemistry study leads to the peer reviewers to be selected based on other expertise. Although the role of TEM is usually minor, it is the only technique that allows native and direct imaging of the sample and has the power to indisputably prove the nature of an assembly and therefore its contribution is much greater. Misinterpretation of TEM data can have a significant impact on the conclusions that are reached. Ice‐contamination in cryo‐TEM as an illustration of the sample, mistaking solid for hollow objects and sample re‐organisation because of drying are but a few examples of mistakes that can lead to erroneous conclusions about systems that are designed for drug‐delivery, catalysis and other important applications. This essay gives a focussed and summarized review of TEM techniques and how to use and interpret them correctly for soft materials.

Furthermore, the ease with which suggestive results can be produced with samples that do not contain any structured materials is demonstrated, providing a strong recommendation to the chemical society to avoid drying and instead use negative stain or preferably cryo‐TEM.

## 2 Techniques

Many electron microscopic sample preparation techniques have been developed since the first electron microscopes became available, each giving different information and requiring different interpretations. To choose the best sample preparation method for soft materials, native representation and sample dehydration play crucial roles. For soft materials like vesicles and proteins, and for the study of phase behaviour, dehydration of the sample should be taken into account as a key factor for proper method selection. In the field of colloid chemistry, several techniques of sample preparation are widely applied: drying, freeze‐drying, freeze fracture, negative or positive stain, embedding followed by sectioning, quick‐freeze deep‐etch and cryo‐TEM.

**Figure 1:**
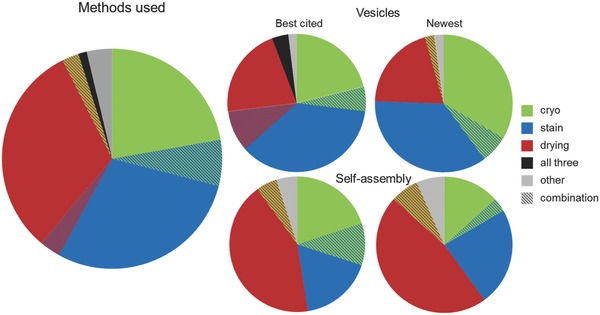
Electron microscopic methods used for the evaluation of “vesicles” and “self‐assembly” structures. Methods of all literature combined (left), and divided into groups of roughly 50 best cited and 50 newest papers of each search (right). Green represents cryo‐TEM, blue staining and red drying, black represents all three of them and grey a different method altogether.

To learn which TEM techniques are most popular in supramolecular chemistry, a systematic literature study composed of two separate searches was conducted in the Web of Science database from 2010 till 2015. Both searches contained “transmission electron microscopy OR TEM” in the topic, one with “vesicle*” and one with “self‐assembly” in the title. We chose to investigate the 50 most cited papers and the 50 newest papers that came out of these searches. Literature that fell outside the range of soft matter chemistry, e.g. cell biology and hard matter, was ignored and partly compensated for by continuing beyond the 50 entries to reach at total of 275 papers. Of the 275 papers 162 contained original TEM data in the field of soft matter or supramolecular chemistry (Tables S1–S4). Relevant literature was sorted by method (**Figure** **1**).

Most papers (156) used drying, staining, cryo‐TEM or a combination of these methods for the analysis of vesicles or self‐assembled structures. Of the analysed papers, 31% dried their samples without any staining, 27% used stain, 23% did cryo‐TEM, 15% applied multiple techniques and the remaining 4% worked with a different technique all together (Figure 1).

Considering the fact that cryo‐TEM images the sample in its most native state, there is a promising increase in the use of cryo‐TEM between best‐cited literature and newest literature about vesicles. Unfortunately this is not the case when searching for self‐assembly. The best cited papers about self‐assembly contained more cryo‐TEM compared to the newest literature (Figure 1). All in all it is disappointing that only 29% of the papers included cryo‐TEM as one of the techniques.

### 2.1 Drying

Drying (including freeze drying) is the most readily accessible and widely applied method to study self‐assembly and vesicles (39%). Typically, a drop of 2–5 µl sample is applied onto a carbon or polymer coated grid and dried for a few minutes up to several hours before imaging.^27^, ^28^ The technique originates from material sciences and is suitable for extremely stable materials.^29^, ^30^, ^31^ Freeze drying, sublimation of surface water and quick‐freeze deep‐etch have the dogma to preserve the structure, but even if the sample is vitrified rapidly, upon warming the sample to –80 °C to remove the water, recrystallization of vitrified water damages and alters the sample if used on colloidal suspensions.^26^, ^32^, ^33^

**Figure 2:**
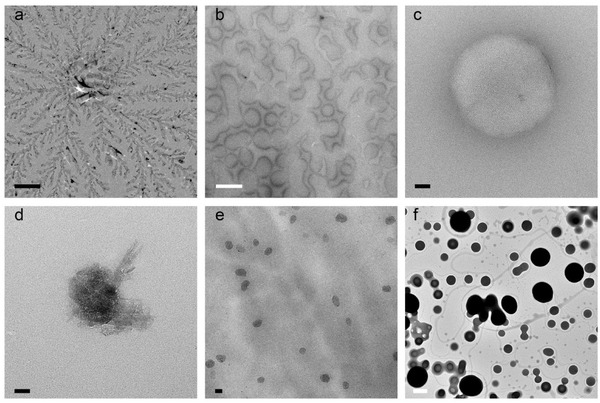
Demonstration of different structures that can be found upon drying of a) a buffer (Hepes, NaCl, α‐Dodecyl Maltoside); various water soluble polymers: b) poly‐(N,N‐dimethylacrylamide (mw 39 kD),^35^ c and d) polystyrene sulfonate sodium salt (mw 220 kD) and e) polyethylene oxide (mw 97kD); and f) a molecular motor in dichloromethane/acetonitrile.^36^ These patterns and structures are not representative for these samples in solution, because none of them form aggregates. Different types of drying patterns can be found for different materials, but also on different areas of the grid (c and d), and when varying the polarity of the surface. Black and white scale bars represent 50 nm and 1 µm respectively.

Although the most widely applied, drying is also the most risky technique for soft materials.^34^ Often, an image of a dried sample is not representative for the sample as it is present in solution. A self‐aggregating sample needs to meet many conditions before being suitable for drying and this is rarely the case. Firstly, one needs to proof that aggregates are formed in solution by dynamic light scattering (DLS) or showing the turbidity of the sample. Aggregates larger than 50 nm will scatter light sufficiently to make the sample turbid. This check is very important, because all solutes present will precipitate upon decreasing solvent content and sometimes aggregate upon drying and cause drying patterns, crystals and other high contrast structures to be visible (**Figure** **2**). After drying, it is no longer possible to conclude whether the aggregates in the image are the result of aggregation behaviour of the sample in solution or of the sample upon drying. A dried sample does therefore not often proofs aggregates in the hydrated state. Following this, it is intrinsic that the sample can only be dried when no other solute materials are present, because it is impossible to distinguish sample from solutes.^16^, ^24^ Furthermore, the aggregates need to be stable and not susceptible to changes in concentration (e.g. micelle to vesicle), because upon drying the concentration increases infinitely when the solvent slowly disappears. Moreover, the interactions within the sample need to be able to compete with interactions that can occur with the (carbon) support layer. Many aggregated or associated materials are poorly preserved and not stable enough to resist deformation or reconformation upon dehydration. For example, lipids which behave like vesicles in solution will form a supported lipid bilayer onto the surface of the grid (Figure 5a,b), and therefore the image is no longer representative for the solution.

To illustrate the dangers of drying and the ease with which suggestive images can be made, several clear solutions that contained only soluble materials were dried (Figure 2). Buffer salts lead to typical drying patterns that are termed fractals or flowers in literature (Figure 2a). Other samples form drying patterns that resemble the use of negative stain and these images are often interpreted as vesicles or micelles (Figure 2b,c). In a Scanning electron microscopy (SEM) image, these objects would look like “rings” or “doughnuts”^21^, ^37^ and are usually called so, but they are often the result of drying. Occasionally, crystallisation of soluble material occurs in a structure that resembles the “dessert‐rose” (Figure 2d). The drying of polymers can result in regularly sized aggregates, sometimes even perfectly round, black balls (Figure 2e,f). These aggregates are also frequently interpreted as micelles or vesicles. Often, a drying pattern is present all over the grid, a seemingly good representation of the sample, but nonetheless a representation after drying. The distinction between before and after drying is regularly overlooked. Other frequently occurring mistakes include the measuring of bilayer‐thickness on the black and white defocus fringes around objects, the deduction that a dense object is hollow when this is not demonstrated by the image, but most frequently images do not allow any conclusive information about the sample as it is in solution, because they show typical drying patterns.

In view of this, drying is highly discouraged for soft materials, especially when solution conditions, like ionic strength, pH and concentration are important. Another technique is necessary to confirm presence of particles in solution. At this point atomic force microscopy or SEM of a dried sample faces exactly the same problems and proves nothing. Even though samples exist that look the same in solution as after drying, the method of drying does not allow this distinction between what structure resulted from the sample and what structure is the result of drying.

### 2.2 Staining

**Figure 3:**
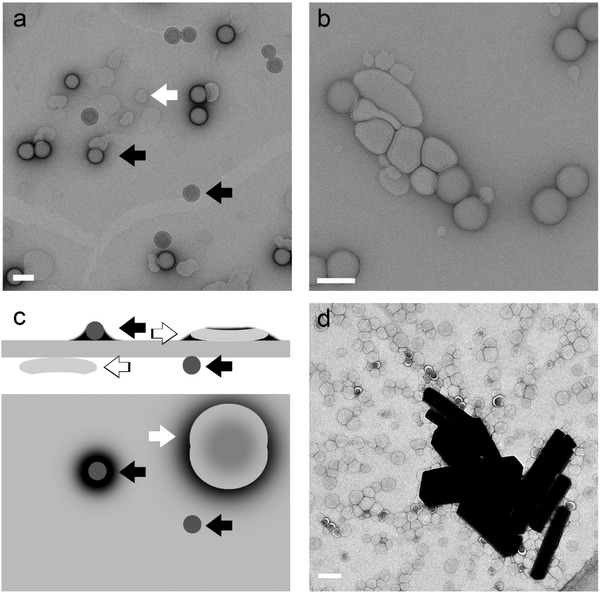
Negative stain EM of phospholipid vesicles (white arrows) mixed with latex spheres (black arrows), negatively stained with a, 2% UAc and b, 2% phospho tungstic acid and schematically visualised with a side‐ and topview (c). Negatively stained objects appear light with a dark halo (arrows). During preparation some sample ended up on the other side of the support grid and was not stained as the UAc solution was only applied to the top of the grid.^39^ Both liposomes and latex are therefore stained and unstained in this image. The unstained liposomes cannot be seen and may be responsible for the contrast differences in the background. The unstained latex (arrow heads) is amorphous, but appears to have some structure inside, this is in fact the imprint into the carbon support film. A similar effect can be sometimes observed in ice contamination. Stained latex can be interpreted as being hollow, but it is not, which becomes clear from the unstained latex. Notice that the intensity of stained and unstained latex is exactly the same. Uranyl acetate crystals deposited on negatively stained liposome sample (d). In absence of sample the crystals can be mistaken for it. Scale bars represent 100 nm.

Negative staining is better suitable for soft matter than drying (**Figure** **3**). In order to preserve a sample and enhance contrast, one can use heavy metals to stain the sample. A positive stain, like for example iodine, ruthenium and osmium tetra oxide,^38^ is a strong scattering agent that adheres to particular areas of the sample. A negative stain does not penetrate the object, but coats the surface and surroundings, obscuring the object itself and all internal structural details, and giving a foot‐print like appearance (Figure 3a,b and d).^39^, ^40^ The most popular stains in soft matter are uranyl acetate (UAc) and phosphotungstic acid (PTA). If the sample does not look negatively stained there is a good chance that the object suffered from dehydration followed by stain absorbance, or that the stain was repelled due to hydrophobicity. In these cases the interpretability of the images will suffer greatly.

Negative stain is applied to the grid straight after the sample was blotted off to prevent dehydration and interactions between the sample and the support grid. Some papers describe the drying of the sample prior to staining, which is incorrect (Figure 1, Figure 5a,b and Figure 6a,b), as the effects of drying may already take place before the nano objects are embedded, and thus fixed by stain.

When choosing to stain, it should be kept in mind what questions can be resolved by this method. Staining is a good method to prove that aggregates exist in solution, but the sample itself is obscured and it is therefore unfeasible to image inner details, like membrane thickness or cargo (Figure 5c). A positive stain could target these, but can change dimensions such as membrane‐thicknesses. It is also impossible to distinguish between disks and spherical objects and rarely possible to differentiate solid and hollow objects unless dehydration causes deformation in a tell‐tale manner, in which case sample dimensions are lost (Figure 5a,b in comparison with Figure 5c,d). Even though the structure is much better preserved upon staining than with drying, upon staining, the sample is nevertheless also dehydrated, which can cause deformation of solvent dependent structures such as lipid vesicles (see Figure 5c in comparison with Figure 5d).

There are many mistakes that can be made with negative staining, starting with incorrect staining. The application has failed when objects appear darker rather than the surrounding and are thus not negatively stained. A good stain shows low contrast objects with a darker halo contouring them. Also with the interpretation of negatively stained images one should take care. Over‐ and misinterpretations include the interpretation of uranyl acetate crystals as the sample (Figure 3d), the deduction that samples are hollow when this does not follow from the image and measuring the membrane thickness on the stain‐layer rather than the actual membrane, which is obscured by the stain.

In order to definitely proof the presence of aggregates and their nature, cryo‐TEM is by far the most native and most informative technique that can be used. Some samples, such as gels and other very viscous materials, cannot easily be frozen for cryo‐TEM. In such a case negative staining is a good alternative, but if possible one should always seek to characterize their supramolecular systems using cryo‐TEM.

### 2.3 Cryo‐TEM

**Figure 4:**
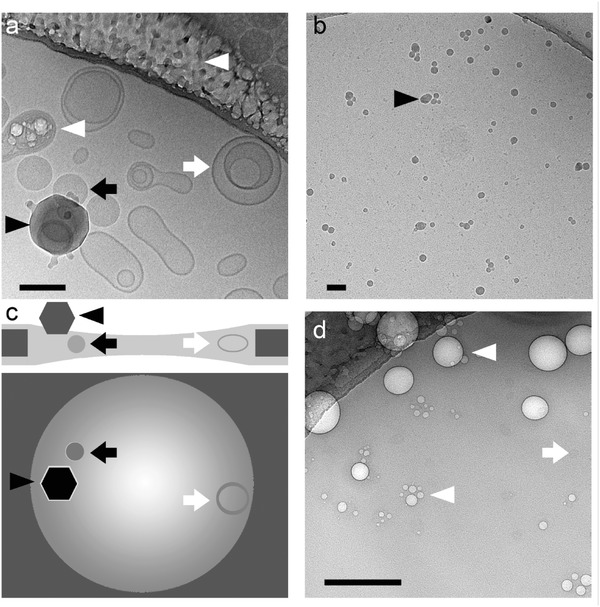
Cryo‐electron microscopy of phospholipid vesicles (white arrows) mixed with latex spheres (black arrows) were plunge‐frozen in liquid ethane (a) and schematically visualised with a side‐ and topview (b). In the schemes, the dark grey is the carbon support and the fading layer is the vitreous ice. As ice contamination (black arrowheads) falls on top of the ice‐layer, it creates extra thickness and has more contrast than latex, which sits inside the vitreous ice. In cryo, the difference between solid and hollow objects can be observed. Figures a and b show ice contamination (black arrow head). Radiation damage (figures a and d, white arrowheads) gives light round bells which can be mistaken for the sample (vaguely visible in the background). Scale bars represent 100 nm.

**Figure 5:**
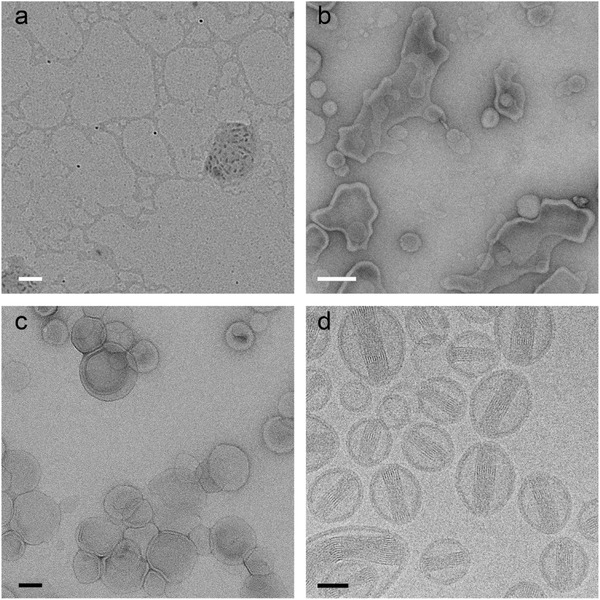
Doxorubicine liposomes (Dox‐NP®) imaged by the most frequently used techniques in soft matter electron microscopy: dried sample without staining (a), UAc stained sample after two minutes of drying (b), negative stained sample (UAc) (c) and cryo‐TEM (d). White scale bars represent 200 nm and black scale bars 50 nm.

**Figure 6:**
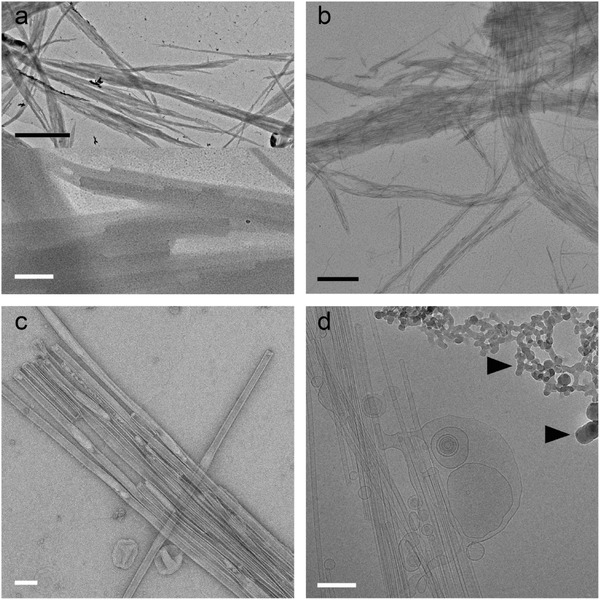
End capped amphiphilic overcrowded alkene nanotubes. a) dried without staining, b) dried before staining, c) negatively stained with 2% UAc and d) cryo‐electron microscopy. In the dried sample only the contours of the nanotubes are visible but the details are completely lost (lower panel of a). The black arrow heads indicate ice contamination. Black scale bars represent 1 µm, white scale bars 200 nm.

In comparison to negative staining, cryo‐TEM images the sample in its most native state (**Figure** **4**a, **Figure** **5**d, **Figure** **6**d). The sample is vitrified in a thin layer of solvent and imaged at very low temperatures, such that the medium neither changes phase nor evaporates in the high vacuum.^41^ The thin layer is made by applying and subsequently blotting a drop of sample on a lacy or holy carbon coated grid. Vitrification of water is achieved by very rapid cooling, usually by plunging the sample into liquid ethane cooled to its melting point.^42^ There is no dehydration, because all procedures are carried out in a humidity controlled environment,^43^, ^44^ which also allows the study of structures at different temperatures. Since the vitrification process is very fast, the sample is physically fixed in its current state.^45^ Where negative stain embeds the structures in the stain, creating a footprint and thus showing the contour and surface of the sample, cryo‐TEM allows visualization through it, making the difference between a hollow object and solids or emulsions apparent (Figure 5d).

Cryo‐TEM images can suffer from hexagonal and vitreous ice contamination. Water, from the humid air, condenses into small particles in the liquid nitrogen and adheres to the grid, giving black ice‐crystals in the images (Figure 4a,b, Figure 6d).^16^, ^24^, ^25^ Leopard skin is vitreous ice, depositing from the vacuum onto the cold surface of the specimen and giving the sample a spotted appearance.^46^ Both ice contamination types as well as radiation damage have high contrast and can be well spread over the sample, which can understandably lead to the incorrect conclusion that this is the sample if the observer is not experienced. Ice contamination can be minimized by drying the liquid nitrogen storage containers before use and keeping strict hygiene measures such as never reuse liquid nitrogen that has been exposed to (humid) air. Other mistakes that are occasionally observed include the misinterpretation of the carbon support background as micelles and even the holes in lacy carbon films for being vesicles. Similar as with drying, the concentric focus fringes around objects, which are caused by defocussing of the objective lens to enhance phase‐contrast, are misinterpreted as bilayers.

Although by far the most suitable technique to study soft matter systems, the number of chemical studies that use cryo‐TEM as a TEM method to study their system is quite limited. Cryo‐TEM is the most straight‐forward and native technique with the least chance of modifying the sample. It will reach the highest resolution and the data, with some knowledge on ice‐contamination, is also the easiest to interpret. Therefore it is quite disappointing that only 29% of the studied literature uses this technique and it is highly recommended that cryo‐TEM becomes the standard technique for soft material characterization.

## 3 Two Soft Matter Samples, Four Preparation Methods

### 3.1 Doxil®

Doxorubicine‐loaded stealth liposomes,^17^ was one of the first liposomal drug formulations that was approved by the FDA for use against certain types of cancer. By encapsulation of the highly toxic doxorubicine in liposomes, side effects were reduced and the effective local dose could be increased. Dox‐NP® (Avanti polar lipids) was imaged with TEM using the four different methods which were found to be most common in soft matter electron microscopy (Figure 1).

Upon drying (Figure 5a), the liposomes and their cargo are lost and show a completely different morphology compared to in solution. When negative staining (2% uranyl acetate, Philips CM120, 120 keV) is applied to a partly dried (3 µl) sample (Figure 5b), as can be occasionally be found described in a materials and methods section, it can be seen that the structure of the liposomes is altered by the drying process, as the lipids started to interact with the carbon to form a supported lipid bilayer. Comparing this to proper staining (Figure 5c) the difference is apparent. The negative stain fixes the structures by embedding them in a glassy environment. The presence of heavy metals give sufficient contrast. Only with cryo‐TEM (Figure 5d), the liposomes were imaged in their native state and the enclosed doxorubicine crystal is visible (FEI Tecnai T20, 200 keV, Gatan model 626 cryo‐stage, low‐dose imaging).

### 3.2 Amphiphilic Nanotubes

Vesicle‐capped nanotubes of an amphiphilic overcrowded alkene mixed with the common phospholipid DOPC were observed with the most popular TEM techniques (Figure 6). Where drying makes the tubes look like fibers, stain after drying enhances this effect. Proper staining with UAc allows beautiful visualisation of the tubes, some of them were even stained on the inside. Still, cryo‐TEM images the sample in its native state. Measuring the ‘bilayer’ in the stained image gives incomparable results to those measured in cryo‐TEM (≈4 nm in comparison to ≈3 nm). Lack of effects of flattening by dehydration and drying patterns of stain, make cryo‐TEM measurements more accurately.

## 4 Quality and Outlook

Placed in a wider perspective against other research fields that use TEM as a characterisation tool, it may be noted that the field of supramolecular chemistry, which is developing rapidly in many areas, seems to be falling behind with respect to the proper use of TEM. Although good studies with beautiful TEM can be found where cryo‐electron microscopy^47^ and 3D methods such as cryo‐electron tomography^19^, ^48^ and 2D^49^ and 3D single‐particle analyses^50^ have been put to use on soft matter structures, too many misapplications and misinterpretations are present in the current literature.

Cryo‐TEM has been developed already in the seventies and eighties^51^, ^52^, ^53^ when it was realized that drying causes many different problems for soft materials and staining can introduce artefacts and is limited because its indirectness.^54^, ^55^ Other research fields that use cryo‐TEM are making more and more use of new technical developments such as the volta‐phase plate^56^ that enhances image contrast and allows imaging in focus. Another great and recent development is the direct detection camera^57^ that induced a true resolution revolution^47^ in the field of structural biology because of its ability to record movies that allow the correction for drift and the detection of radiation damage that accumulates with exposure time.

In summary, drying of soft materials is often unjustified and the misinterpretation of dried materials is a persisting problem. The instances that drying of soft matter is without consequence are rare. In many cases, the structures cannot be directly related to the state of aggregation in solution. Cryo‐TEM is much more suited, and therefore we urge that drying of soft matter is no longer accepted as prove of aggregation. Some samples, such as very viscous materials like gels cannot easily be frozen, and for those negative staining is a good alternative, but, if possible, one should always seek to characterize supramolecular systems using cryo‐TEM. Soft matter chemistry studies can gain a wealth of new and in depth information about their systems when the field starts to use cryo‐TEM and makes use of the applications and software that have been developed in other fields.

## Supporting information

As a service to our authors and readers, this journal provides supporting information supplied by the authors. Such materials are peer reviewed and may be re‐organized for online delivery, but are not copy‐edited or typeset. Technical support issues arising from supporting information (other than missing files) should be addressed to the authors.

Supplementary
