# Supplementary material for: Transmission Electron Microscopy as a Tool for the Characterization of Soft Materials: Application and Interpretation
Source: Adv Sci (Weinh). 2017 Jan 31;4(5):1600476. doi: 10.1002/advs.201600476 (PMC5441488; doi:10.1002/advs.201600476)
Supplement: Supplementary file 1 — Supplementary [file ADVS-4-na-s001.pdf]

Copyright WILEY-VCH Verlag GmbH & Co. KGaA, 69469 Weinheim, Germany, 2017.

Supporting Information

for *Adv. Sci.,* DOI: 10.1002/advs.201600476

Transmission Electron Microscopy as a Tool for the Characterization of Soft Materials: Application and Interpretation

*Linda E. Franken, Egbert J. Boekema, and Marc C. A. Stuart**

Transmission Electron Microscopy as a Tool for the Characterization of Soft Materials: Application and Interpretation

Linda E. Franken[1], Egbert J. Boekema[1] and Marc C.A. Stuart[1,2]*

[1] Electron microscopy Group, Groningen Biomolecular Sciences and Biotechnology Institute and [2] Stratingh Institute for Chemistry, University of Groningen, Nijenborgh 7, 9747 AG Groningen, The Netherlands

Reference information used for the statistical analysis on application per category. The duplicates that are found between the different searches were counted only once in the overall statistics.

Table S1. Vesicles best cited 2010-2015

Reference	Method
1	Stain
2	Drying
3	Drying
4	Stain
5	Stain (after drying)
6	Drying
7	Cryo
8	Stain (after drying)
9	Stain, cryo
10	Cryo
11	Stain
12	Stain
13	Stain
14	Cryo
15	Drying
16	Drying, stain
17	Stain, cryo
18	Drying
19	Drying
20	Drying
21	Stain (after drying)
22	Stain (after drying), cryo
23	Drying, stain
24	Drying
25	Freeze fracture
26	Cryo
27	Stain
28	Stain
29	Drying

Reference	Method
30	Stain
31	Drying, stain, cryo
32	Drying, stain, freeze fracture
33	Cryo
34	Stain
35	Cryo
36	Stain
37	Drying
38	Stain (after drying)
39	Cryo
40	Cryo
41	Cryo
42	Drying, stain (after drying)
43	Stain
44	Drying
45	Drying, stain
46	Cryo
47	Drying, stain, cryo
48	Stain
49	Stain
50	Cryo
51	Stain
52	Stain

Table S2. Vesicles newest 2015

Reference	Method
53	Cryo
54	Stain
55	Stain
56	Cryo
57	Stain
58	Stain
59	Drying
60	Stain
61	Drying, cryo
62	Freeze fracture
63	Stain (after drying)
64	Cryo
65	Cryo
66	Stain (after drying), cryo
67	Cryo
68	Drying
69	Cryo
70	Drying
71	Stain, drying
72	Cryo
73	Stain
74	Cryo
75	Cryo
76	Drying
77	Drying
78	Stain
79	Drying
80	Stain (after drying)
81	Cryo
82	Drying
83	Stain, cryo
84	Stain
85	Cryo
86	Cryo
87	Stain
88	Stain
89	Drying
90	Stain
91	Cryo
92	Cryo
93	Stain
94	Stain
95	Stain, cryo
96	Drying
97	Cryo

Table S3. Self-assembly best cited 2010-2015

Reference	Method
98	Cryo
99	Drying, sectioning
100	Drying
101	Other
102	Stain, cryo
103	Drying
104	Drying
105	Stain, cryo
106	Stain, cryo
5	Stain (after drying)
107	Stain
108	Stain, cryo
109	Drying
110	Cryo
7	Cryo
111	Drying
112	Stain
113	Drying
114	Drying
115	Stain
116	Drying
117	Drying
118	Cryo
119	Cryo
120	Drying
121	Drying
122	Drying, stain, cryo
123	Cryo
14	Cryo
124	Other
125	Drying, cryo
126	Drying
127	Drying
128	Cryo
129	Drying
130	Drying
131	Drying
4	Stain
132	Stain
133	Stain

Table S4. Self-assembly newest 2015

Reference	Method
134	Drying
135	Drying
136	Drying
137	Stain
138	Stain
139	Stain
140	Cryo
141	Stain
142	Embedding/sectioning
143	Cryo
144	Cryo
145	Stain
146	Drying
61	Drying, cryo
147	Drying
148	Drying
149	Drying
150	Drying
151	Drying
152	Stain
153	Drying
154	Drying
155	Drying
156	Drying, cryo
157	Embedding/sectioning
158	Stain, cryo
159	Drying
160	Cryo
161	Stain
162	Drying

References

(1) Blanazs, A.; Madsen, J.; Battaglia, G.; Ryan, A. J.; Armes, S. P. Mechanistic Insights for Block Copolymer Morphologies: How do Worms Form Vesicles? *J. Am. Chem. Soc.* **2011***, 133*, 16581-16587.

(2) Duan, Q.; Cao, Y.; Li, Y.; Hu, X.; Xiao, T.; Lin, C.; Pan, Y.; Wang, L. pH-Responsive Supramolecular Vesicles Based on Water-Soluble Pillar[6]Arene and Ferrocene Derivative for Drug Delivery. *J. Am. Chem. Soc.* **2013***, 135*, 10542-10549.

(3) Wang, K.; Guo, D.; Wang, X.; Liu, Y. Multistimuli Responsive Supramolecular Vesicles Based on the Recognition of P-Sulfonatocalixarene and its Controllable Release of Doxorubicin. *Acs Nano* **2011***, 5*, 2880-2894.

(4) Wang, Y.; Han, P.; Xu, H.; Wang, Z.; Zhang, X.; Kabanov, A. V. Photocontrolled Self-Assembly and Disassembly of Block Ionomer Complex Vesicles: A Facile Approach Toward Supramolecular Polymer Nanocontainers. *Langmuir* **2010***, 26*, 709-715.

(5) Smith, A. E.; Xu, X.; Kirkland-York, S. E.; Savin, D. A.; McCormick, C. L. "Schizophrenic" Self-Assembly of Block Copolymers Synthesized Via Aqueous RAFT Polymerization: From Micelles to Vesicles. *Macromolecules* **2010***, 43*, 1210-1217.

(6) Mai, Y.; Eisenberg, A. Controlled Incorporation of Particles into the Central Portion of Vesicle Walls. *J. Am. Chem. Soc.* **2010***, 132*, 10078-10084.

(7) Rasch, M. R.; Rossinyol, E.; Hueso, J. L.; Goodfellow, B. W.; Arbiol, J.; Korgel, B. A. Hydrophobic Gold Nanoparticle Self-Assembly with Phosphatidylcholine Lipid: Membrane-Loaded and Janus Vesicles. *Nano Lett.* **2010***, 10*, 3733-3739.

(8) Moughton, A. O.; O'Reilly, R. K. Thermally Induced Micelle to Vesicle Morphology Transition for a Charged Chain End Diblock Copolymer. *Chem. Commun.* **2010***, 46*, 1091-1093.

(9) Wang, C.; Chen, Q.; Xu, H.; Wang, Z.; Zhang, X. Photoresponsive Supramolecular Amphiphiles for Controlled Self-Assembly of Nanofibers and Vesicles. *Adv Mater* **2010***, 22*, 2553-2555.

(10) Voskuhl, J.; Stuart, M. C. A.; Ravoo, B. J. Sugar-Decorated Sugar Vesicles: Lectin-Carbohydrate Recognition at the Surface of Cyclodextrin Vesicles. *Chem. -Eur. J.* **2010***, 16*, 2790-2796.

(11) Chambon, P.; Blanazs, A.; Battaglia, G.; Armes, S. P. Facile Synthesis of Methacrylic ABC Triblock Copolymer Vesicles by RAFT Aqueous Dispersion Polymerization. *Macromolecules* **2012***, 45*, 5081-5090.

(12) Fielding, L. A.; Derry, M. J.; Ladmiral, V.; Rosselgong, J.; Rodrigues, A. M.; Ratcliffe, L. P. D.; Sugihara, S.; Armes, S. P. RAFT Dispersion Polymerization in Non-Polar Solvents: Facile Production of Block Copolymer Spheres, Worms and Vesicles in N-Alkanes. *Chem. Sci.* **2013***, 4*, 2081-2087.

(13) Chen, D. J.; Osterrieder, N.; Metzger, S. M.; Buckles, E.; Doody, A. M.; DeLisa, M. P.; Putnam, D. Delivery of Foreign Antigens by Engineered Outer Membrane Vesicle Vaccines. *Proc. Natl. Acad. Sci. U. S. A.* **2010***, 107*, 3099-3104.

(14) Pietsch, C.; Mansfeld, U.; Guerrero-Sanchez, C.; Hoeppener, S.; Vollrath, A.; Wagner, M.; Hoogenboom, R.; Saubern, S.; Thang, S. H.; Becer, C. R.; Chiefari, J.; Schubert, U. S. Thermo-Induced Self-Assembly of Responsive Poly(DMAEMA-B-DEGMA) Block Copolymers into Multi- and Unilamellar Vesicles. *Macromolecules* **2012***, 45*, 9292-9302.

(15) Bo, X.; Bai, J.; Yang, L.; Guo, L. The Nanocomposite of PtPd Nanoparticles/Onion-Like Mesoporous Carbon Vesicle for Nonenzymatic Amperometric Sensing of Glucose. *Sens. Actuator B-Chem.* **2011***, 157*, 662-668.

(16) Yuan, J.; Bai, X.; Zhao, M.; Zheng, L. C(12)mimBr Ionic Liquid/SDS Vesicle Formation and use as Template for the Synthesis of Hollow Silica Spheres. *Langmuir* **2010***, 26*, 11726-11731.

(17) Park, K. M.; Lee, D.; Sarkar, B.; Jung, H.; Kim, J.; Ko, Y. H.; Lee, K. E.; Jeon, H.; Kim, K. Reduction-Sensitive, Robust Vesicles with a Non-Covalently Modifiable Surface as a Multifunctional Drug-Delivery Platform. *Small* **2010***, 6*, 1430-1441.

(18) Huang, C.; Pan, C. Direct Preparation of Vesicles from One-Pot RAFT Dispersion Polymerization. *Polymer* **2010***, 51*, 5115-5121.

(19) Du, J.; Fan, L.; Liu, Q. pH-Sensitive Block Copolymer Vesicles with Variable Trigger Points for Drug Delivery. *Macromolecules* **2012***, 45*, 8275-8283.

(20) Liu, G.; Jin, Q.; Liu, X.; Lv, L.; Chen, C.; Ji, J. Biocompatible Vesicles Based on PEO-B-PMPC/Alpha-Cyclodextrin Inclusion Complexes for Drug Delivery. *Soft Matter* **2011***, 7*, 662-669.

(21) Chen, W.; Du, J. Ultrasound and pH Dually Responsive Polymer Vesicles for Anticancer Drug Delivery. *Sci. Rep.* **2013***, 3*, 2162.

(22) Wang, T.; Jiang, J.; Liu, Y.; Li, Z.; Liu, M. Hierarchical Self-Assembly of Bolaamphiphiles with a Hybrid Spacer and L-Glutamic Acid Headgroup: pH- and Surface-Triggered Hydrogels, Vesicles, Nanofibers, and Nanotubes. *Langmuir* **2010***, 26*, 18694-18700.

(23) Ren, T.; Liu, Q.; Lu, H.; Liu, H.; Zhang, X.; Du, J. Multifunctional Polymer Vesicles for Ultrasensitive Magnetic Resonance Imaging and Drug Delivery. *J. Mater. Chem.* **2012***, 22*, 12329-12338.

(24) Zhang, J.; Song, Y.; Cronin, L.; Liu, T. Reverse-Vesicle Formation of Organic-Inorganic Polyoxometalate-Containing Hybrid Surfactants with Tunable Sizes. *Chem. -Eur. J.* **2010***, 16*, 11320-11324.

(25) Marianecci, C.; Paolino, D.; Celia, C.; Fresta, M.; Carafa, M.; Alhaique, F. Non-Ionic Surfactant Vesicles in Pulmonary Glucocorticoid Delivery: Characterization and Interaction with Human Lung Fibroblasts. *J. Controlled Release* **2010***, 147*, 127-135.

(26) Thiele, J.; Steinhauser, D.; Pfohl, T.; Foerster, S. Preparation of Monodisperse Block Copolymer Vesicles Via Flow Focusing in Microfluidics. *Langmuir* **2010***, 26*, 6860-6863.

(27) Sun, T.; Guo, Q.; Zhang, C.; Hao, J.; Xing, P.; Su, J.; Li, S.; Hao, A.; Liu, G. Self-Assembled Vesicles Prepared from Amphiphilic Cyclodextrins as Drug Carriers. *Langmuir* **2012***, 28*, 8625-8636.

(28) Bhosale, S. V.; Jani, C. H.; Lalander, C. H.; Langford, S. J.; Nerush, I.; Shapter, J. G.; Villamaina, D.; Vauthey, E. Supramolecular Construction of Vesicles Based on Core-Substituted Naphthalene Diimide Appended with Triethyleneglycol Motifs. *Chem. Commun.* **2011***, 47*, 8226-8228.

(29) Azzam, T.; Eisenberg, A. Fully Collapsed (Kippah) Vesicles: Preparation and Characterization. *Langmuir* **2010***, 26*, 10513-10523.

(30) Zhang, H.; An, W.; Liu, Z.; Hao, A.; Hao, J.; Shen, J.; Zhao, X.; Sun, H.; Sun, L. Redox-Responsive Vesicles Prepared from Supramolecular Cyclodextrin Amphiphiles. *Carbohydr. Res.* **2010***, 345*, 87-96.

(31) Du, J.; O'Reilly, R. K. pH-Responsive Vesicles from a Schizophrenic Diblock Copolymer. *Macromol. Chem. Phys.* **2010***, 211*, 1530-1537.

(32) Martin, L.; Castro, E.; Ribeiro, A.; Alonso, M.; Rodriguez-Cabello, J. C. Temperature-Triggered Self-Assembly of Elastin-Like Block Co-Recombinamers: The Controlled Formation of Micelles and Vesicles in an Aqueous Medium. *Biomacromolecules* **2012***, 13*, 293-298.

(33) Wang, D.; Dong, R.; Long, P.; Hao, J. Photo-Induced Phase Transition from Multilamellar Vesicles to Wormlike Micelles. *Soft Matter* **2011***, 7*, 10713-10719.

(34) Zhao, W.; Chen, D.; Hu, Y.; Grason, G. M.; Russell, T. P. ABC Triblock Copolymer Vesicles with Mesh-Like Morphology. *Acs Nano* **2011***, 5*, 486-492.

(35) Bauduin, P.; Prevost, S.; Farras, P.; Teixidor, F.; Diat, O.; Zemb, T. A Theta-Shaped Amphiphilic Cobaltabisdicarbollide Anion: Transition from Monolayer Vesicles to Micelles. *Angew. Chem. Int. Edit.* **2011***, 50*, 5298-5300.

(36) Chessa, M.; Caddeo, C.; Valenti, D.; Manconi, M.; Sinico, C.; Fadda, A. M. Effect of Penetration Enhancer Containing Vesicles on the Percutaneous Delivery of Quercetin through New Born Pig Skin. *Pharmaceutics* **2011***, 3*, 497-509.

(37) Gao, L.; Zheng, B.; Yao, Y.; Huang, F. Responsive Reverse Giant Vesicles and Gel from Self-Organization of a Bolaamphiphilic Pillar[5]Arene. *Soft Matter* **2013***, 9*, 7314-7319.

(38) Ghosh, S.; Dey, J. Interaction of Sodium N-Lauroylsarcosinate with N-Alkylpyridinium Chloride Surfactants: Spontaneous Formation of pH-Responsive, Stable Vesicles in Aqueous Mixtures. *J. Colloid Interf. Sci.* **2011***, 358*, 208-216.

(39) Mizuno, N.; Varkey, J.; Kegulian, N. C.; Hegde, B. G.; Cheng, N.; Langen, R.; Steven, A. C. Remodeling of Lipid Vesicles into Cylindrical Micelles by Alpha-Synuclein in an Extended Alpha-Helical Conformation. *J. Biol. Chem.* **2012***, 287*, 29301-29311.

(40) Malsam, J.; Parisotto, D.; Bharat, T. A. M.; Scheutzow, A.; Krause, J. M.; Briggs, J. A. G.; Soellner, T. H. Complexin Arrests a Pool of Docked Vesicles for Fast Ca2+-Dependent Release. *EMBO J.* **2012***, 31*, 3270-3281.

(41) Voskuhl, J.; Fenske, T.; Stuart, M. C. A.; Wibbeling, B.; Schmuck, C.; Ravoo, B. J. Molecular Recognition of Vesicles: Host-Guest Interactions Combined with Specific Dimerization of Zwitterions. *Chem. -Eur. J.* **2010***, 16*, 8300-8306.

(42) Zhu, Y.; Fan, L.; Yang, B.; Du, J. Multifunctional Homopolymer Vesicles for Facile Immobilization of Gold Nanoparticles and Effective Water Remediation. *Acs Nano* **2014***, 8*, 5022-5031.

(43) Ghosh, S.; Ghatak, C.; Banerjee, C.; Mandal, S.; Kuchlyan, J.; Sarkar, N. Spontaneous Transition of Micelle-Vesicle-Micelle in a Mixture of Cationic Surfactant and Anionic Surfactant-Like Ionic Liquid: A Pure Nonlipid Small Unilamellar Vesicular Template used for Solvent and Rotational Relaxation Study. *Langmuir* **2013***, 29*, 10066-10076.

(44) Jin, Q.; Luy, C.; Ji, J.; Agarwal, S. Design and Proof of Reversible Micelle-to-Vesicle Multistimuli-Responsive Morphological Regulations. *J. Polym. Sci. Pol. Chem.* **2012***, 50*, 451-457.

(45) Lu, H.; Fan, L.; Liu, Q.; Wei, J.; Ren, T.; Du, J. Preparation of Water-Dispersible Silver-Decorated Polymer Vesicles and Micelles with Excellent Antibacterial Efficacy. *Polym. Chem.* **2012***, 3*, 2217-2227.

(46) Wu, C.; Hu, Y.; Deng, M.; Huang, X.; Yu, D.; Xiang, J.; Liu, Y.; Li, Z.; Wang, Y. Molecular Conformation-Controlled Vesicle/Micelle Transition of Cationic Trimeric Surfactants in Aqueous Solution. *Langmuir* **2010***, 26*, 7922-7927.

(47) Castelletto, V.; Cheng, G.; Stain, C.; Connon, C. J.; Hamley, I. W. Self-Assembly of a Peptide Amphiphile Containing L-Carnosine and its Mixtures with a Multilamellar Vesicle Forming Lipid. *Langmuir* **2012***, 28*, 11599-11608.

(48) Sun, T.; Li, Y.; Zhang, H.; Li, J.; Xin, F.; Kong, L.; Hao, A. pH-Reversible Vesicles Based on the "Supramolecular Amphiphilies" Formed by Cyclodextrin and Anthraquinone Derivate. *Colloid Surf. A-Physicochem. Eng. Asp.* **2011***, 375*, 87-96.

(49) Zhang, K.; Wang, G.; Zhao, X.; Jiang, X.; Li, Z. Vesicle Self-Assembly by Tetrathiafulvalene Derivatives in both Polar and Nonpolar Solvents and Pseudo-Rotaxane Mediated Vesicle-to-Microtube Transformation. *Langmuir* **2010***, 26*, 6878-6882.

(50) Yuk, S. H.; Oh, K. S.; Koo, H.; Jeon, H.; Kim, K.; Kwon, I. C. Multi-Core Vesicle Nanoparticles Based on Vesicle Fusion for Delivery of Chemotherapic Drugs. *Biomaterials* **2011***, 32*, 7924-7931.

(51) Sun, T.; Zhang, H.; Kong, L.; Qiao, H.; Li, Y.; Kin, F.; Hao, A. Controlled Transformation from Nanorods to Vesicles Induced by Cyclomaltoheptaoses (Beta-Cyclodextrins). *Carbohydr. Res.* **2011***, 346*, 285-293.

(52) Zhang, H.; Sun, L.; Liu, Z.; An, W.; Hao, A.; Xin, F.; Shen, J. pH-Responsive Vesicle-Like Particles Based on Inclusion Complexes between Cyclodextrins and Methyl Orange. *Colloid Surf. A-Physicochem. Eng. Asp.* **2010***, 358*, 115-121.

(53) Manca, M. L.; Castangia, I.; Zaru, M.; Nacher, A.; Valenti, D.; Fernandez-Busquets, X.; Fadda, A. M.; Manconi, M. Development of Curcumin Loaded Sodium Hyaluronate Immobilized Vesicles (Hyalurosomes) and their Potential on Skin Inflammation and Wound Restoring. *Biomaterials* **2015***, 71*, 100-109.

(54) Dong, S.; Spicer, P. T.; Lucien, F. P.; Zetterlund, P. B. Synthesis of Crosslinked Polymeric Nanocapsules using Catanionic Vesicle Templates Stabilized by Compressed CO2. *Soft Matter* **2015***, 11*, 8613-8620.

(55) Wang, J.; Chen, X.; Cui, W.; Yi, S. pH-Responsive Vesicles from Supra-Amphiphiles Based on Dynamic Imine Bond. *Colloid Surf. A-Physicochem. Eng. Asp.* **2015***, 484*, 28-36.

(56) Jiang, Z.; Jia, K.; Liu, X.; Dong, J.; Li, X. Multiple Responsive Fluids Based on Vesicle to Wormlike Micelle Transitions by Single-Tailed Pyrrolidone Surfactants. *Langmuir* **2015***, 31*, 11760-11768.

(57) Vezocnik, V.; Rebolj, K.; Sitar, S.; Ota, K.; Tusek-Znidaric, M.; Strus, J.; Sepcic, K.; Pahovnik, D.; Macek, P.; Zagar, E. Size Fractionation and Size Characterization of Nanoemulsions of Lipid Droplets and Large Unilamellar Lipid Vesicles by Asymmetric-Flow Field-Flow Fractionation/Multi-Angle Light Scattering and Dynamic Light Scattering. *J. Chromatogr. A* **2015***, 1418*, 185-191.

(58) Alenaizi, R.; Radiman, S.; Rahman, I. A.; Mohamed, F. Zwitterionic Betaine-Cholesterol System: Effects of Sonication Duration and Aging on Vesicles Stability. *Colloid Surf. A-Physicochem. Eng. Asp.* **2015***, 482*, 662-669.

(59) Menon, S.; Ongungal, R. M.; Das, S. Vesicle-to-Rod Transition of Polymer Aggregates upon Irradiation. *Macromol. Chem. Phys.* **2015***, 216*, 1997-2006.

(60) Zheng, Z.; Liu, C.; Qiao, W. pH-Responsive and CO2-Responsive Vesicles can be Formed by N-Decylimidazole. *Eur. J. Lipid Sci. Technol.* **2015***, 117*, 1673-1678.

(61) He, Q.; Ao, Y.; Huang, Z.; Wang, D. Self-Assembly and Disassembly of Vesicles as Controlled by Anion-Pi Interactions. *Angew Chem Int Edit* **2015***, 54*, 11785-11790.

(62) Zhang, J.; Xu, G.; Song, A.; Wang, L.; Lin, M.; Dong, Z.; Yang, Z. Faceted Fatty Acid Vesicles Formed from Single-Tailed Perfluorinated Surfactants. *Soft Matter* **2015***, 11*, 7143-7150.

(63) Pan, A.; Rakshit, S.; Sahu, S.; Bhattacharya, S. C.; Moulik, S. P. Synergism between Anionic Double Tail and Zwitterionic Single Tail Surfactants in the Formation of Mixed Micelles and Vesicles, and use of the Micelle Templates for the Synthesis of Nano-Structured Gold Particles. *Colloid Surf. A-Physicochem. Eng. Asp.* **2015***, 481*, 644-654.

(64) de Souza, T. P.; Holzer, M.; Stano, P.; Steiniger, F.; May, S.; Schubert, R.; Fahr, A.; Luisi, P. L. New Insights into the Growth and Transformation of Vesicles: A Free-Flow Electrophoresis Study. *J Phys Chem B* **2015***, 119*, 12212-12223.

(65) Uda, R. M.; Nishimoto, N.; Yamamoto, M. Inter-Vesicle Polymerization using Nonionic Oxyethylene-Hydrogenated Castor Oil. *Colloid Surf. A-Physicochem. Eng. Asp.* **2015***, 480*, 45-49.

(66) Chen, Y.; Zhang, K.; Wang, X.; Zhang, F.; Zhu, J.; Mays, J. W.; Wooley, K. L.; Pochan, D. J. Multigeometry Nanoparticles: Hybrid Vesicle/Cylinder Nanoparticles Constructed with Block Copolymer Solution Assembly and Kinetic Control. *Macromolecules* **2015***, 48*, 5621-5631.

(67) Castangia, I.; Manca, M. L.; Caddeo, C.; Maxia, A.; Murgia, S.; Pons, R.; Demurtas, D.; Pando, D.; Falconieri, D.; Peris, J. E.; Fadda, A. M.; Manconi, M. Faceted Phospholipid Vesicles Tailored for the Delivery of Santolina Insularis Essential Oil to the Skin. *Colloid Surf. B-Biointerfaces* **2015***, 132*, 185-193.

(68) Yu, J.; Zhang, Y.; Ye, Y.; DiSanto, R.; Sun, W.; Ranson, D.; Ligler, F. S.; Buse, J. B.; Gu, Z. Microneedle-Array Patches Loaded with Hypoxia-Sensitive Vesicles Provide Fast Glucose-Responsive Insulin Delivery. *Proc. Natl. Acad. Sci. U. S. A.* **2015***, 112*, 8260-8265.

(69) Kuchlyan, J.; Banik, D.; Roy, A.; Kundu, N.; Sarkar, N. Vesicles Formation by Zwitterionic Micelle and Poly-L-Lysine: Solvation and Rotational Relaxation Study. *J Phys Chem B* **2015***, 119*, 8285-8292.

(70) Ioannou, C. P.; Ioannou, G. I.; Moushi, E. E.; Velonia, K.; Chronakis, N. Self-Assembled Giant Vesicles Formed by Type I [3:3]-Hexakis Adducts of C-60 Equipped with Enantiomerically Pure Cyclo-Monomalonate Addends. *Eur. J. Org. Chem.* **2015**, 4598-4602.

(71) Qin, J.; Liu, Q.; Zhang, J.; Chen, J.; Chen, S.; Zhao, Y.; Du, J. Rationally Separating the Corona and Membrane Functions of Polymer Vesicles for Enhanced T-2 MRI and Drug Delivery. *ACS Appl. Mater. Interfaces* **2015***, 7*, 14043-14052.

(72) Caddeo, C.; Valenti, D.; Nacher, A.; Manconi, M.; Fadda, A. M. Exploring the Co-Loading of Lidocaine Chemical Forms in Surfactant/Phospholipid Vesicles for Improved Skin Delivery. *J. Pharm. Pharmacol.* **2015***, 67*, 909-917.

(73) Li, C.; Zhang, S.; Pang, J.; Wu, Y.; Gu, Z. Facile Fabrication of Robust Organic Counterion-Induced Vesicles: Reversible Thermal Behavior for Optical Temperature Sensor and Synergistic Catalyst upon Removal of Amine. *Adv. Funct. Mater.* **2015***, 25*, 3764-3774.

(74) Xing, L.; Wang, X.; Gao, X.; Chen, B.; Tung, C.; Wu, L. Self-Assembled Vesicles from Amphiphilic Platinum(II) Terpyridyl Complex in Water. *Supramol. Chem.* **2015***, 27*, 298-302.

(75) Zhao, W.; Feng, L.; Xu, L.; Xu, W.; Sun, X.; Hao, J. Chiroptical Vesicles and Disks that Originated from Achiral Molecules. *Langmuir* **2015***, 31*, 5748-5757.

(76) Artetxe, B.; Reinoso, S.; San Felices, L.; Gutierrez-Zorrilla, J. M.; Garcia, J. A.; Haso, F.; Liu, T.; Vicent, C. Crown-Shaped Tungstogermanates as Solvent-Controlled Dual Systems in the Formation of Vesicle-Like Assemblies. *Chem-Eur J* **2015***, 21*, 7736-+.

(77) Li, W.; Luo, T.; Yang, Y.; Tan, X.; Liu, L. Formation of Controllable Hydrophilic/Hydrophobic Drug Delivery Systems by Electrospinning of Vesicles. *Langmuir* **2015***, 31*, 5141-5146.

(78) Caddeo, C.; Manconi, M.; Cardia, M. C.; Diez-Sales, O.; Fadda, A. M.; Sinico, C. Investigating the Interactions of Resveratrol with Phospholipid Vesicle Bilayer and the Skin: NMR Studies and Confocal Imaging. *Int. J. Pharm.* **2015***, 484*, 138-145.

(79) Yang, H.; Zhang, C.; Li, C.; Liu, Y.; An, Y.; Ma, R.; Shi, L. Glucose-Responsive Polymer Vesicles Templated by Alpha-CD/PEG Inclusion Complex. *Biomacromolecules* **2015***, 16*, 1372-1381.

(80) Guo, F.; Wang, J.; Ma, M.; Tan, F.; Li, N. Skin Targeted Lipid Vesicles as Novel Nano-Carrier of Ketoconazole: Characterization, in Vitro and in Vivo Evaluation. *J. Mater. Sci. -Mater. Med.* **2015***, 26*, 175.

(81) Rao, K. S.; Gehlot, P. S.; Gupta, H.; Drechsler, M.; Kumar, A. Sodium Bromide Induced Micelle to Vesicle Transitions of Newly Synthesized Anionic Surface Active Ionic Liquids Based on Dodecylbenzenesulfonate. *J Phys Chem B* **2015***, 119*, 4263-4274.

(82) Zhao, J.; Zhang, H.; Sun, H.; Liu, Y. Supramolecular Nanoassemblies of an Amphiphilic Porphyrin-Cyclodextrin Conjugate and their Morphological Transition from Vesicle to Network. *Chem. -Eur. J.* **2015***, 21*, 4457-4464.

(83) Li, S.; Zhang, L.; Wang, B.; Ma, M.; Xing, P.; Chu, X.; Zhang, Y.; Hao, A. An Easy Approach for Constructing Vesicles by using Aromatic Molecules with Beta-Cyclodextrin. *Soft Matter* **2015***, 11*, 1767-1777.

(84) Yu, Y.; Li, J.; Zhang, M.; Cao, L.; Isaacs, L. Hydrophobic Monofunctionalized Cucurbit[7]Uril Undergoes Self-Inclusion Complexation and Forms Vesicle-Type Assemblies. *Chem. Commun.* **2015***, 51*, 3762-3765.

(85) Sun, N.; Shi, L.; Lu, F.; Xie, S.; Sun, P.; Zheng, L. Spontaneous Vesicle Phase Formation by Linear Pseudo-Oligomeric Surfactant in Aqueous Solutions. *Langmuir* **2015***, 31*, 2281-2287.

(86) Silva, S. G.; do Vale, M. L. C.; Marques, E. F. Size, Charge, and Stability of Fully Serine-Based Catanionic Vesicles: Towards Versatile Biocompatible Nanocarriers. *Chem. -Eur. J.* **2015***, 21*, 4092-4101.

(87) Ma, M.; Xu, S.; Xing, P.; Li, S.; Chu, X.; Hao, A. A Multistimuli-Responsive Supramolecular Vesicle Constructed by Cyclodextrins and Tyrosine. *Colloid Polym. Sci.* **2015***, 293*, 891-900.

(88) Micheletto, Y. M. S.; da Silveira, N. P.; Barboza, D. M.; dos Santos, M. C.; de Lima, V. R.; Giacomelli, F. C.; Martinez, J. C. V.; Frizon, T. E. A.; Dal Bo, A. G. Investigation of Self-Association between New Glycosurfactant N-Acetyl-Beta-D-Glucosaminyl-PEG-Docosanate and Soybean Phosphatidylcholine into Vesicles. *Colloid Surf. A-Physicochem. Eng. Asp.* **2015***, 467*, 166-172.

(89) Li, G.; Qi, M.; Yu, N.; Liu, X. Hybrid Vesicles Co-Assembled from Anionic Graft Copolymer and Metal Ions for Controlled Drug Release. *Chem. Eng. J.* **2015***, 262*, 710-715.

(90) Warren, N. J.; Mykhaylyk, O. O.; Ryan, A. J.; Williams, M.; Doussineau, T.; Dugourd, P.; Antoine, R.; Portale, G.; Armes, S. P. Testing the Vesicular Morphology to Destruction: Birth and Death of Diblock Copolymer Vesicles Prepared Via Polymerization-Induced Self-Assembly. *J. Am. Chem. Soc.* **2015***, 137*, 1929-1937.

(91) Dao, T. P. T.; Fernandes, F.; Er-Rafik, M.; Salva, R.; Schmutz, M.; Brulet, A.; Prieto, M.; Sandre, O.; Le Meins, J. -. Phase Separation and Nanodomain Formation in Hybrid Polymer/Lipid Vesicles. *ACS Macro Lett.* **2015***, 4*, 182-186.

(92) Drazenovic, J.; Wang, H.; Roth, K.; Zhang, J.; Ahmed, S.; Chen, Y.; Bothun, G.; Wunder, S. L. Effect of Lamellarity and Size on Calorimetric Phase Transitions in Single Component Phosphatidylcholine Vesicles. *Biochim. Biophys. Acta-Biomembr.* **2015***, 1848*, 532-543.

(93) Ma, M.; Shang, W.; Xing, P.; Li, S.; Chu, X.; Hao, A.; Liu, G.; Zhang, Y. A Supramolecular Vesicle of Camptothecin for its Water Dispersion and Controllable Releasing. *Carbohydr. Res.* **2015***, 402*, 208-214.

(94) Zhu, W.; Fang, S.; Zhang, Y.; Li, X. Functional Vesicles Formed by Anticancer Drug Assembly. *Bioorg. Med. Chem. Lett.* **2015***, 25*, 188-191.

(95) Maiti, C.; Banerjee, R.; Maiti, S.; Dhara, D. pH-Induced Vesicle-to-Micelle Transition in Amphiphilic Diblock Copolymer: Investigation by Energy Transfer between in Situ Formed Polymer Embedded Gold Nanoparticles and Fluorescent Dye. *Langmuir* **2015***, 31*, 32-41.

(96) Chen, L.; He, H.; Jin, Y. Counting and Dynamic Studies of the Small Unilamellar Phospholipid Vesicle Trans Location with Single Conical Glass Nanopores. *Anal. Chem.* **2015**, *87*, 522-529.

(97) Patel, V.; Ray, D.; Singh, K.; Abezgauz, L.; Marangoni, G.; Aswal, V. K.; Bahadur, P. 1-Hexanol Triggered Structural Characterization of the Worm-Like Micelle to Vesicle Transitions in Cetyltrimethylammonium Tosylate Solutions. *Rsc Advances* **2015**, *5*, 87758-87768.

(98) Percec, V., et al Self-Assembly of Janus Dendrimers into Uniform Dendrimersomes and Other Complex Architectures. *Science* **2010**, *328*, 1009-1014.

(99) Yao, Y.; Xue, M.; Chen, J.; Zhang, M.; Huang, F. An Amphiphilic Pillar[5]Arene: Synthesis, Controllable Self-Assembly in Water, and Application in Calcein Release and TNT Adsorption. *J. Am. Chem. Soc.* **2012**, *134*, 15712-15715.

(100) Takacs, C. J.; Sun, Y.; Welch, G. C.; Perez, L. A.; Liu, X.; Wen, W.; Bazan, G. C.; Heeger, A. J. Solar Cell Efficiency, Self-Assembly, and Dipole-Dipole Interactions of Isomorphic Narrow-Band-Gap Molecules. *J. Am. Chem. Soc.* **2012**, *134*, 16597-16606.

(101) Chuvilin, A.; Bichoutskaia, E.; Gimenez-Lopez, M. C.; Chamberlain, T. W.; Rance, G. A.; Kuganathan, N.; Biskupek, J.; Kaiser, U.; Khlobystov, A. N. Self-Assembly of a Sulphur-Terminated Graphene Nanoribbon within a Single-Walled Carbon Nanotube. *Nat. Mater.* **2011**, *10*, 687-692.

(102) Petzetakis, N.; Dove, A. P.; O'Reilly, R. K. Cylindrical Micelles from the Living Crystallization-Driven Self-Assembly of Poly(Lactide)-Containing Block Copolymers. *Chem. Sci.* **2011**, *2*, 955-960.

(103) Yallapu, M. M.; Jaggi, M.; Chauhan, S. C. Beta-Cyclodextrin-Curcumin Self-Assembly Enhances Curcumin Delivery in Prostate Cancer Cells. *Colloid Surf. B-Biointerfaces* **2010**, *79*, 113-125.

(104) Yu, G.; Ma, Y.; Han, C.; Yao, Y.; Tang, G.; Mao, Z.; Gao, C.; Huang, F. A Sugar-Functionalized Amphiphilic Pillar[5]Arene: Synthesis, Self-Assembly in Water, and Application in Bacterial Cell Agglutination. *J. Am. Chem. Soc.* **2013**, *135*, 10310-10313.

(105) Ziserman, L.; Lee, H.; Raghavan, S. R.; Mor, A.; Danino, D. Unraveling the Mechanism of Nanotube Formation by Chiral Self-Assembly of Amphiphiles. *J. Am. Chem. Soc.* **2011**, *133*, 2511-2517.

(106) del Barrio, J.; Oriol, L.; Sanchez, C.; Luis Serrano, J.; Di Cicco, A.; Keller, P.; Li, M. Self-Assembly of Linear-Dendritic Diblock Copolymers: From Nanofibers to Polymersomes. *J. Am. Chem. Soc.* **2010**, *132*, 3762-3769.

(107) Gou, P.; Zhu, W.; Shen, Z. Synthesis, Self-Assembly, and Drug-Loading Capacity of Well-Defined Cyclodextrin-Centered Drug-Conjugated Amphiphilic A(14)B(7) Miktoarm Star Copolymers Based on Poly(Epsilon-Caprolactone) and Poly(Ethylene Glycol). *Biomacromolecules* **2010**, *11*, 934-943.

(108) Groeger, G.; Meyer-Zaika, W.; Boettcher, C.; Groehn, F.; Ruthard, C.; Schmuck, C. Switchable Supramolecular Polymers from the Self-Assembly of a Small Monomer with Two Orthogonal Binding Interactions. *J. Am. Chem. Soc.* **2011**, *133*, 8961-8971.

(109) Xia, J.; Yin, S.; Li, H.; Xu, H.; Yan, Y.; Zhang, Q. Self-Assembly and Enhanced Photocatalytic Properties of BiOI Hollow Microspheres Via a Reactable Ionic Liquid. *Langmuir* **2011**, *27*, 1200-1206.

(110) Sun, R.; Xue, C.; Ma, X.; Gao, M.; Tian, H.; Li, Q. Light-Driven Linear Helical Supramolecular Polymer Formed by Molecular-Recognition-Directed Self-Assembly of Bis(P-Sulfonatocalix[4]Arene) and Pseudorotaxane. *J. Am. Chem. Soc.* **2013***, 135*, 5990-5993.

(111) Agut, W.; Brulet, A.; Schatz, C.; Taton, D.; Lecommandoux, S. pH and Temperature Responsive Polymeric Micelles and Polymersomes by Self-Assembly of Poly[2-(Dimethylamino)Ethyl Methacrylate]-B-Poly(Glutamic Acid) Double Hydrophilic Block Copolymers. *Langmuir* **2010***, 26*, 10546-10554.

(112) Zhang, Z.; Liu, K. L.; Li, J. Self-Assembly and Micellization of a Dual Thermoresponsive Supramolecular Pseudo-Block Copolymer. *Macromolecules* **2011***, 44*, 1182-1193.

(113) Gudarzi, M. M.; Sharif, F. Self Assembly of Graphene Oxide at the Liquid-Liquid Interface: A New Route to the Fabrication of Graphene Based Composites. *Soft Matter* **2011***, 7*, 3432-3440.

(114) Yao, Y.; Xue, M.; Zhang, Z.; Zhang, M.; Wang, Y.; Huang, F. Gold Nanoparticles Stabilized by an Amphiphilic Pillar[5]Arene: Preparation, Self-Assembly into Composite Microtubes in Water and Application in Green Catalysis. *Chem. Sci.* **2013***, 4*, 3667-3672.

(115) Tang, C.; Ulijn, R. V.; Saiani, A. Effect of Glycine Substitution on Fmoc-Diphenylalanine Self-Assembly and Gelation Properties. *Langmuir* **2011***, 27*, 14438-14449.

(116) Si, W.; Hu, X.; Liu, X.; Fan, R.; Chen, Z.; Weng, L.; Hou, J. Self-Assembly and Proton Conductance of Organic Nanotubes from Pillar[5]Arenes. *Tetrahedron Lett.* **2011***, 52*, 2484-2487.

(117) Jiang, B.; Guo, D.; Liu, Y. Self-Assembly of Amphiphilic Perylene-Cyclodextrin Conjugate and Vapor Sensing for Organic Amines. *J. Org. Chem.* **2010***, 75*, 7258-7264.

(118) Tidhar, Y.; Weissman, H.; Wolf, S. G.; Gulino, A.; Rybtchinski, B. Pathway-Dependent Self-Assembly of Perylene Diimide/Peptide Conjugates in Aqueous Medium. *Chem. -Eur. J.* **2011***, 17*, 6068-6075.

(119) Skrabania, K.; von Berlepsch, H.; Boettcher, C.; Laschewsky, A. Synthesis of Ternary, Hydrophilic-Lipophilic-Fluorophilic Block Copolymers by Consecutive RAFT Polymerizations and their Self-Assembly into Multicompartment Micelles. *Macromolecules* **2010***, 43*, 271-281.

(120) Chen, Q.; Feng, Y.; Zhang, D.; Zhang, G.; Fan, Q.; Sun, S.; Zhu, D. Light-Triggered Self-Assembly of a Spiropyran-Functionalized Dendron into Nano-/Micrometer-Sized Particles and Photoresponsive Organogel with Switchable Fluorescence. *Adv. Funct. Mater.* **2010***, 20*, 36-42.

(121) Po, C.; Tam, A. Y.; Wong, K. M.; Yam, V. W. Supramolecular Self-Assembly of Amphiphilic Anionic Platinum(II) Complexes: A Correlation between Spectroscopic and Morphological Properties. *J. Am. Chem. Soc.* **2011***, 133*, 12136-12143.

(122) Kim, D.; Kim, E.; Lee, J.; Hong, S.; Sung, W.; Lim, N.; Park, C. G.; Kim, K. Direct Synthesis of Polymer Nanocapsules: Self-Assembly of Polymer Hollow Spheres through Irreversible Covalent Bond Formation. *J. Am. Chem. Soc.* **2010***, 132*, 9908-9919.

(123) Percec, V., et al Modular Synthesis of Amphiphilic Janus Glycodendrimers and their Self-Assembly into Glycodendrimersomes and Other Complex Architectures with Bioactivity to Biomedically Relevant Lectins. *J. Am. Chem. Soc.* **2013***, 135*, 9055-9077.

(124) Johnson, E. K.; Adams, D. J.; Cameron, P. J. Directed Self-Assembly of Dipeptides to Form Ultrathin Hydrogel Membranes. *J. Am. Chem. Soc.* **2010***, 132*, 5130-5136.

(125) Neelakandan, P. P.; Pan, Z.; Hariharan, M.; Zheng, Y.; Weissman, H.; Rybtchinski, B.; Lewis, F. D. Hydrophobic Self-Assembly of a Perylenediimide-Linked DNA Dumbbell into Supramolecular Polymers. *J. Am. Chem. Soc.* **2010***, 132*, 15808-15813.

(126) Duan, P.; Qin, L.; Zhu, X.; Liu, M. Hierarchical Self-Assembly of Amphiphilic Peptide Dendrons: Evolution of Diverse Chiral Nanostructures through Hydrogel Formation Over a Wide pH Range. *Chem. -Eur. J.* **2011***, 17*, 6389-6395.

(127) Zhang, W.; He, J.; Liu, Z.; Ni, P.; Zhu, X. Biocompatible and pH-Responsive Triblock Copolymer mPEG-B-PCL-B-PDMAEMA: Synthesis, Self-Assembly, and Application. *J. Polym. Sci. Pol. Chem.* **2010***, 48*, 1079-1091.

(128) Marsat, J.; Heydenreich, M.; Kleinpeter, E.; Berlepsch, H. V.; Boettcher, C.; Laschewsky, A. Self-Assembly into Multicompartment Micelles and Selective Solubilization by Hydrophilic-Lipophilic-Fluorophilic Block Copolymers. *Macromolecules* **2011***, 44*, 2092-2105.

(129) Yu, X.; Zhang, W.; Yue, K.; Li, X.; Liu, H.; Xin, Y.; Wang, C.; Wesdemiotis, C.; Cheng, S. Z. D. Giant Molecular Shape Amphiphiles Based on Polystyrene-Hydrophilic [60]Fullerene Conjugates: Click Synthesis, Solution Self-Assembly, and Phase Behavior. *J. Am. Chem. Soc.* **2012***, 134*, 7780-7787.

(130) Jin, Q.; Liu, G.; Liu, X.; Ji, J. Photo-Responsive Supramolecular Self-Assembly and Disassembly of an Azobenzene-Containing Block Copolymer. *Soft Matter* **2010***, 6*, 5589-5595.

(131) Ortel, E.; Reier, T.; Strasser, P.; Kraehnert, R. Mesoporous IrO2 Films Templated by PEO-PB-PEO Block-Copolymers: Self-Assembly, Crystallization Behavior, and Electrocatalytic Performance. *Chem. Mat.* **2011***, 23*, 3201-3209.

(132) Bowerman, C. J.; Nilsson, B. L. A Reductive Trigger for Peptide Self-Assembly and Hydrogelation. *J. Am. Chem. Soc.* **2010***, 132*, 9526-9527.

(133) Chen, L.; Morris, K.; Laybourn, A.; Elias, D.; Hicks, M. R.; Rodger, A.; Serpell, L.; Adams, D. J. Self-Assembly Mechanism for a Naphthalene-Dipeptide Leading to Hydrogelation. *Langmuir* **2010***, 26*, 5232-5242.

(134) Hedir, G. G.; Pitto-Barry, A.; Dove, A. P.; O'Reilly, R. K. Amphiphilic Block Copolymer Self-Assemblies of Poly(NVP)-B-Poly(MDO-Co-Vinyl Esters): Tunable Dimensions and Functionalities. *J. Polym. Sci. Pol. Chem.* **2015***, 53*, 2699-2710.

(135) Wang, G.; Yuan, D.; Yuan, T.; Dong, J.; Feng, N.; Han, G. A Visible Light Responsive Azobenzene-Functionalized Polymer: Synthesis, Self-Assembly, and Photoresponsive Properties. *J. Polym. Sci. Pol. Chem.* **2015***, 53*, 2768-2775.

(136) Zhang, C.; Yang, S.; Zhu, Y.; Zhang, R.; Liu, X. Formation of Bowl-Shaped Nanoparticles by Self-Assembly of Cinnamic Acid-Modified Dextran. *Carbohydr. Polym.* **2015***, 133*, 637-643.

(137) Huang, Y.; Li, L.; Li, G. An Enzyme-Catalysed Access to Amphiphilic Triblock Copolymer of PCL-B-PEG-B-PCL: Synthesis, Characterization and Self-Assembly Properties. *Des. Monomers Polym.* **2015***, 18*, 799-806.

(138) Vilaca, H.; Hortelao, A. C. L.; Castanheira, E. M. S.; Queiroz, M. R. P.; Hilliou, L.; Hamley, I. W.; Martins, J. A.; Ferreira, P. M. T. Dehydrodipeptide Hydrogelators Containing Naproxen N-Capped Tryptophan: Self-

Assembly, Hydrogel Characterization, and Evaluation as Potential Drug Nanocarriers. *Biomacromolecules* **2015**, *16*, 3562-3573.

(139) Bengani, P.; Kou, Y.; Asatekin, A. Zwitterionic Copolymer Self-Assembly for Fouling Resistant, High Flux Membranes with Size-Based Small Molecule Selectivity. *J. Membr. Sci.* **2015**, *493*, 755-765.

(140) Li, Y.; Li, H.; Chai, J.; Chen, M.; Yang, Q.; Hao, J. Self-Assembly and Rheological Properties of a Pseudogemini Surfactant Formed in a Salt-Free Catanionic Surfactant Mixture in Water. *Langmuir* **2015**, *31*, 11209-11219.

(141) Pei, Y.; Noy, J.; Roth, P. J.; Lowe, A. B. Soft Matter Nanoparticles with Reactive Coronal Pentafluorophenyl Methacrylate Residues Via Non-Polar RAFT Dispersion Polymerization and Polymerization-Induced Self-Assembly. *J. Polym. Sci. Pol. Chem.* **2015**, *53*, 2326-2335.

(142) Madhavan, P.; Sougrat, R.; Behzad, A. R.; Peinemann, K.; Nunes, S. P. Ionic Liquids as Self-Assembly Guide for the Formation of Nanostructured Block Copolymer Membranes. *J. Membr. Sci.* **2015**, *492*, 568-577.

(143) Deng, Y.; Zou, T.; Tao, X.; Semetey, V.; Trepout, S.; Marco, S.; Ling, J.; Li, M. Poly(Epsilon-Caprolactone)-Block-Polysarcosine by Ring-Opening Polymerization of Sarcosine N-Thiocarboxyanhydride: Synthesis and Thermoresponsive Self-Assembly. *Biomacromolecules* **2015**, *16*, 3265-3274.

(144) Nummelin, S.; Liljestrom, V.; Saarikoski, E.; Ropponen, J.; Nykanen, A.; Linko, V.; Seppala, J.; Hirvonen, J.; Ikkala, O.; Bimbo, L. M.; Kostiainen, M. A. Self-Assembly of Amphiphilic Janus Dendrimers into Mechanically Robust Supramolecular Hydrogels for Sustained Drug Release. *Chem. -Eur. J.* **2015**, *21*, 14433-14439.

(145) Zhang, W.; Bao, H.; He, J.; Dong, X. Preparation of Multicompartment Micelles from Amphiphilic Linear Triblock Terpolymers by pH-Responsive Self-Assembly. *Colloid Polym. Sci.* **2015**, *293*, 3013-3024.

(146) Bhosale, R. S.; Al Kobaisi, M.; Bhosale, S. V.; Bhargava, S.; Bhosale, S. V. Flower-Like Supramolecular Self-Assembly of Phosphonic Acid Appended Naphthalene Diimide and Melamine. *Sci. Rep.* **2015**, *5*, 14609.

(147) Pawar, G. M.; Lalancette, R. A.; Bonder, E. M.; Sheridan, J. B.; Jaekle, F. ROMP-Derived Pyridylborate Block Copolymers: Self-Assembly, pH-Responsive Properties, and Metal-Containing Nanostructures. *Macromolecules* **2015**, *48*, 6508-6515.

(148) Chen, L.; Ren, Y.; Wu, N.; Sun, B.; Ma, J.; Zhang, L.; Tan, H.; Liu, M.; Li, X.; Yang, H. Hierarchical Self-Assembly of Discrete Organoplatinum(II) Metallacycles with Polysaccharide Via Electrostatic Interactions and their Application for Heparin Detection. *J. Am. Chem. Soc.* **2015**, *137*, 11725-11735.

(149) Tyagi, M.; Kartha, K. P. R. Synthesis of Glycotriazololipids and Observations on their Self-Assembly Properties. *Carbohydr. Res.* **2015**, *413*, 85-92.

(150) Xue, X.; Yang, J.; Huang, W.; Yang, H.; Jiang, B.; Li, F.; Jiang, Y. Dual Thermo- and Light-Responsive Nanorods from Self-Assembly of the 4-Propoxyazobenzene-Terminated Poly(N-Isopropylacrylamide) in Aqueous Solution. *Polymer* **2015**, *73*, 195-204.

(151) Yang, P.; Li, C.; Chen, H.; Juang, R. Synthesis, Photochemical Properties, and Self-Assembly of Diblock Copolymer Bearing Azobenzene Moieties. *J. Taiwan Inst. Chem. Eng.* **2015**, *54*, 155-164.

(152) Allievi Frizon, T. E.; Serafini Micheletto, Y. M.; Westrup, J. L.; Silva Wakabayashi, P. S.; Serafim, F. R.; Damiani, A. P.; Longaretti, L. M.; de Andrade, V. M.; Giacomelli, F. C.; Fort, S.; Dal Bo, A. G. New Glycosylated Conjugate

Copolymer N-Acetyl-Beta-D-Glucosaminyl-Pluronic: Synthesis, Self-Assembly and Biological Assays. *Colloid Surf. B-Biointerfaces* **2015***, 133*, 323-330.

(153) Liang, J.; Wu, W.; Li, J.; Han, C.; Zhang, S.; Guo, J.; Zhou, H. Synthesis and Self-Assembly of Temperature and Anion Double Responsive Ionic Liquid Block Copolymers. *Front. Mater. Sci.* **2015***, 9*, 254-263.

(154) Jie, K.; Zhou, Y.; Yao, Y.; Shi, B.; Huang, F. CO2-Responsive Pillar[5]Arene-Based Molecular Recognition in Water: Establishment and Application in Gas-Controlled Self-Assembly and Release. *J. Am. Chem. Soc.* **2015***, 137*, 10472-10475.

(155) Chen, J.; Guo, B.; Eyster, T. W.; Ma, P. X. Super Stretchable Electroactive Elastomer Formation Driven by Aniline Trimer Self-Assembly. *Chem. Mat.* **2015***, 27*, 5668-5677.

(156) Menard-Moyon, C.; Venkatesh, V.; Krishna, K. V.; Bonachera, F.; Verma, S.; Bianco, A. Self-Assembly of Tyrosine into Controlled Supramolecular Nanostructures. *Chem. -Eur. J.* **2015***, 21*, 11681-11686.

(157) Kim, H.; Kim, Y.; Cho, S.; Lee, M. Self-Assembly of a Tripod Aromatic Rod into Stacked Planar Networks. *Chem. -Eur. J.* **2015***, 21*, 11836-11842.

(158) Fu, J.; Guerette, P. A.; Miserez, A. Self-Assembly of Recombinant Hagfish Thread Keratins Amenable to a Strain-Induced Alpha-Helix to Beta-Sheet Transition. *Biomacromolecules* **2015***, 16*, 2327-2339.

(159) Ma, S.; Zhou, J.; Wali, A. R. M.; He, Y.; Xu, X.; Tang, J. Z.; Gu, Z. Self-Assembly of pH-Sensitive Fluorinated Peptide Dendron Functionalized Dextran Nanoparticles for on-Demand Intracellular Drug Delivery. *J. Mater. Sci. -Mater. Med.* **2015***, 26*, 219.

(160) Khimani, M.; Yusa, S.; Nagae, A.; Enomoto, R.; Aswal, V. K.; Kesselman, E.; Danino, D.; Bahadur, P. Self-Assembly of Multi-Responsive Poly(N-Isopropylacrylamide)-B-Poly(N,N-Dimethylaminopropylacrylamide) in Aqueous Media. *Eur. Polym. J.* **2015***, 69*, 96-109.

(161) Roy, A.; Maiti, M.; Roy, S. Effect of Hydrocarbon Chain and Amide Linkage on the Interfacial and Self-Assembly Properties of Nicotinic Acid Amphiphiles. *J. Chem. Eng. Data* **2015***, 60*, 2209-2218.

(162) Shi, S.; He, Y.; Chen, W.; Liu, N.; Zhu, Y.; Ding, Y.; Yin, J.; Wu, Z. Polypeptide-B-Poly(Phenyl Isocyanide) Hybrid Rod-Rod Copolymers: One-Pot Synthesis, Self-Assembly, and Cell Imaging. *Macromol. Rapid Commun.* **2015***, 36*, 1511-1520.
